# Nurses' Self-Efficacy, Job Embeddedness, and Psychological Empowerment: A Cross-Sectional Study

**DOI:** 10.1155/jonm/6259635

**Published:** 2025-04-08

**Authors:** Xin Wang, Ming Liu, Angela Y. M. Leung, Jun-E Zhang, Renli Deng, Yan Li, Yan Wang, Hongxia Dai, Xiaoyan Jin, Shaomei Shang

**Affiliations:** ^1^Faculty of Health Sciences and Sports, Macao Polytechnic University, Macao, China; ^2^Peking University Health Science Center-Macao Polytechnic University Nursing Academy, Macao Polytechnic University, Macao, China; ^3^School of Nursing, The Hong Kong Polytechnic University, Hong Kong, China; ^4^WHO Collaborating Centre for Community Health Services, The Hong Kong Polytechnic University, Hong Kong, China; ^5^Research Institute of Smart Ageing (RISA), The Hong Kong Polytechnic University, Hong Kong, China; ^6^School of Nursing, Sun Yat-Sen University, Guangzhou, China; ^7^Department of Nursing, Zunyi Medical University, Zunyi, China; ^8^School of Nursing, Peking University, Beijing, China

**Keywords:** directed acyclic graph, job embeddedness, nurses, psychological empowerment, self-efficacy

## Abstract

**Background:** To strengthen and motivate the nursing workforce, this study explored the relationship between nurses' self-efficacy, job embeddedness, and psychological empowerment, and how this relationship varied across three regions in the Guangdong–Hong Kong–Macao Greater Bay Area of China.

**Methods:** A multicenter cross-sectional study surveyed 3806 nurses between March and July 2023 using the Sociodemographic Information Questionnaire, Self-Efficacy Scale, Job Embeddedness Scale, and Psychological Empowerment Scale. A directed acyclic graph was used to expose the minimum sufficient adjustment sets for the influence hypothesized model, which was used as a covariate in the model. Pearson correlation analysis, multiple linear regression, and mediation effect analysis were used to test the relationship between variables. The moderated mediation model was employed to test the moderating effect of regions.

**Results:** The psychological empowerment score of 3806 participants was medium-high level (45.22 ± 6.89); self-efficacy (*B* = 0.642, *p* < 0.001) and job embeddedness (*B* = 0.189, *p* < 0.001) directly affected psychological empowerment. Job embeddedness mediated self-efficacy and psychological empowerment (*B* = 0.300, 95% CI: [0.266, 0.355]), but there was no indirect association between self-efficacy and psychological empowerment among Hong Kong participants (*B* = 0.024, 95% CI: [−0.079, 0.150]). Specifically, regions of Guangdong–Hong Kong moderated the relationship between self-efficacy and job embeddedness (*B* = −1.447, *p* < 0.001), and self-efficacy was not significantly associated with job embeddedness (*B* = 0.147, *p*=0.539) among Hong Kong nurses.

**Conclusion:** Managers should acknowledge the influence and significance of nurses in the current healthcare environment. By truly enhancing nurses' psychological empowerment, organizations can foster a genuine sense of empowerment, thereby promoting nurse leadership and improving nurse retention. Improving nurses' self-efficacy can increase job embeddedness and further increase psychological empowerment. This model needs further validation in regions with different cultural and societal backgrounds. Future interventions can be made by identifying work scenarios that affect nurses' self-efficacy, providing information on self-efficacy and increasing nurses' job embeddedness, which may help to improve their psychological empowerment.

## 1. Introduction

“Nursing is the backbone of health care systems worldwide contributing to better health outcomes, economic savings, and more stable societies [1].” This appeal calls for renewed appreciation of the value of nursing: in view of the aging population, pandemics, and other public health challenges, nurses play a critical role. However, it is estimated that there are 29 million nurses and 2.2 million midwives globally, with a projected shortage of 4.8 million nurses and midwives by 2030 [2]. The worldwide shortage of nursing professionals poses a significant challenge to the sustainability of healthcare systems globally. Given the current growth in demand for health services and the goal of achieving health for all, the recovery and retention of the nursing workforce is an essential prerequisite for the rebuilding of the global health system [3]. Previous studies have identified psychological empowerment as a major factor influencing nurses' leadership [4, 5] and retention intentions [6, 7]. Psychological empowerment is a motivational concept of self-efficacy, defined as a construct of intrinsic motivation and positive orientation in response to work, manifested in four cognitions: meaning, competence, self-determination, and impact [8]. Moreover, the positive effects of psychological empowerment in nursing practice and management continue to be demonstrated [9, 10], and it is crucial to enhance the psychological empowerment of nurses.

## 2. Background

Power and empowerment are of significant importance within the field of nursing practice. Meanwhile, they are complex concepts and exist in a variety of forms and definitions across different theoretical contexts [8, 11–13]. Critical social theory, which focuses on enabling disenfranchised members to overcome domination [14]; organizational theory, which is concerned with the top–down distribution of power in organizations, known as structural empowerment [15]; and poststructuralism, which is concerned with disciplinary power and knowledge/power [16] The obvious difference between other theories and psychological empowerment is that the latter concerns the psychological condition inherent in the employee/individual. This concept defines empowerment from the perspective of the individual, based on social psychological theory, which is seen as a process of personal growth and development [17, 18]. Psychological empowerment represents a significant internal motivational factor, with individual behavior influenced by individual motivation, and organizational-level empowerment measures may not necessarily directly lead to the organizationally desired employee behaviors [10]. Therefore, it is essential for managers to prioritize the psychological experience of those who are empowered to increase their agency. In other words, empowerment only works when employees feel empowered [17].

**Figure 1:**
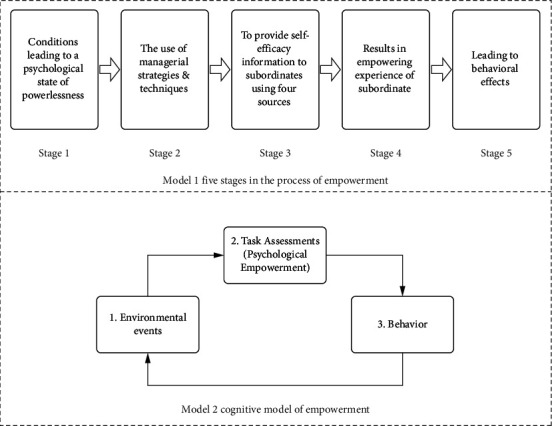
Psychological empowerment model.

While the significance of psychological empowerment in nursing practice is well-established, existing studies predominantly emphasize the empowering behaviors and practices of managers. These include organizational structural change, improvements in communication channels, and employee involvement in decision-making processes [19–22]. However, there remains a notable gap in empirical studies addressing how to enhance nurses' psychological empowerment through targeted interventions. Furthermore, the factors affecting psychological empowerment require further exploration. In accordance with the five stages in the process of empowerment by Conger and Kanungo [17] (Figure 1), the provision of information on self-efficacy to the subordinate (Stage 3) will result in the subsequent experience of empowerment (Stage 4). Self-efficacy refers to an individual's confidence or belief in their ability to achieve behavioral goals in a particular area [23]. The social cognitive theory identifies that self-efficacy is the foundation of an individual's motivation and influences outcome behaviors and expectations [24]. Therefore, it can be considered that when nurses have a high degree of self-efficacy, they will experience higher levels of initiative and intrinsic motivation, which will lead to feeling empowered. Conversely, when nurses lack self-efficacy, they lack motivation and are unable to feel empowered even when managers provide them with empowerment strategies.

Thomas and Velthouse [18] conceptualized empowerment and proposed a cognitive empowerment model (Figure 1), identifying the following problems with Conger and Kanungo's model. First, it is not complete in its conceptual and content structure of empowerment and only considers the concept of self-efficacy. Furthermore, psychological empowerment is not a stable, widespread, cross-situational personality trait but rather a set of cognitions developed in a particular work situation [8]. Therefore, individuals' psychological empowerment level varies with the work situation. The cognitive empowerment model further develops the concept of empowerment, which is centered on a continuous cycle of environmental events, task evaluation (psychological empowerment), and behavior, and emphasizes the importance of the work situation for psychological empowerment. Consequently, the role of environmental events in the influence of self-efficacy on psychological empowerment needs to be considered. Environmental events are defined as the sources of data about the consequences of the individual's ongoing behavior and about conditions relevant to that person's future behavior [18]. It can be interpreted as the result that occurs when an employee performs an action or completes a task, and this result must be assessed as progress or setback and given an interpretation meaning regarding the individual's goals and activities. Job embeddedness is a significant environmental event, which is defined as the broader influence on an employee's decision to continue working. It is also considered a net to capture the combined forces that keep employees embedded in their jobs [25, 26]. Mitchell et al. [25] proposed job embeddedness containing organization (on-the-job) and community (off-the-job) embeddedness and included three work-related forces: link, fit, and sacrifice. When nurses are embedded in their work, they develop a stronger link to the organization, become more fit with the job and function, and perceive the sacrifices it takes to leave the organization. All of this may motivate nurses and help them to perceive being empowered.

In addition, the effect of self-efficacy on job embeddedness has been confirmed, with nurses with high self-efficacy more likely to be embedded in their jobs [27, 28]. Specifically, self-efficacy encourages nurses to cope positively with the tasks and challenges they face in their work and demonstrate greater job satisfaction and professionalism, which helps to improve nurses' job embeddedness [28, 29]. Job embeddedness represents the attractiveness of this job for the nurse, and this attractiveness will hopefully further help nurses with self-efficacy to increase their level of psychological empowerment. Therefore, job embeddedness may play a mediating role in the relationship between self-efficacy and psychological empowerment. Based on the above theoretical framework and evidence, this study aims to validate the effects of nurses' self-efficacy and job embeddedness on psychological empowerment and the mediating role of job embeddedness in the relationship between self-efficacy and psychological empowerment. More specifically, the following hypotheses were formulated:

  Hypothesis 1: Self-efficacy, job embeddedness, and psychological empowerment are positively correlated.
  Hypothesis 2: Self-efficacy and job embeddedness influence psychological empowerment.
  Hypothesis 3: Job embeddedness is a mediating variable between self-efficacy and psychological empowerment.

The study was conducted in the Guangdong–Hong Kong–Macao Greater Bay Area, which includes Guangdong Province in mainland China, as well as the Hong Kong and Macao Special Administrative Regions. These three regions have distinct cultural, economic, and policy backgrounds. Hong Kong, as an international financial center, blends both eastern and western cultures, and Macao's culture is a coexistence culture that is predominantly Chinese and compatible with Portuguese culture. The three regions also have different policies and regulations for the management and training of nurses. Examining the differences in culture and policies among these regions allows us to assess the variations in the proposed models across the three regions, thereby validating the models' cross-cultural adaptability. Specifically, the region-based subgroup analyses shed light on the robustness of the model across cultures, provide insights for proposing and implementing future interventions, and facilitate the generalization of the study's findings across different cultures and regions.

Meanwhile, in order to control for the effect of covariates on the model and obtain accurate estimates of the relationship between these variables, we adapted the model through the directed acyclic graph (DAG) construction of the minimum sufficient adjustment sets (MSASs) to explore the effect of nurses' self-efficacy and job embeddedness on psychological empowerment.

## 3. Methods

### 3.1. Study Design, Setting, and Participants

This multicenter cross-sectional study was conducted from March 2023 to July 2023 in 31 hospitals and 6 health centers in 9 cities and 2 Special Administrative Regions of the Guangdong–Hong Kong–Macao Greater Bay Area, with a total of 37 study settings using the convenience sampling method. The study population consisted of registered and enrolled nurses who were licensed and working full-time in Mainland China, Hong Kong, or Macao Special Administrative Regions. Nurses who did not participate directly in patient care, including those on sick leave, personal leave, or further study, were excluded. The sample size was determined using the Kendall sample estimation method, which recommends a sample size of 5–10 times the number of variables [30]. This study utilized 10 sociodemographic variables and 13 scale and dimension-related variables. To account for a potential 20% invalid response rate, the final minimum sample size calculated was 138–276. The reporting of the study followed the Strengthening the Reporting of Observational Studies in Epidemiology statement for cross-sectional studies [31].

### 3.2. Measurement

#### 3.2.1. Sociodemographic Information Questionnaire

Sociodemographic information includes gender, age, region, marital status, education, monthly salary level, nature of work organization, whether or not you have specialist nurse qualifications, professional title, and working years.

#### 3.2.2. Self-Efficacy Scale (SES)

Developed by Schwarzer [32], a German clinical psychologist, the Chinese version of the SES was translated by Zhang and Schwarzer [33] in 1995 and has supportable reliability and validity. The scale consists of 10 entries and is scored on a Likert 4-point scale (1 = completely incorrect and 4 = completely correct), with scores ranging from 4 to 40, and the higher the score, the higher the level of self-efficacy. Cronbach's *α* in our study was 0.911.

#### 3.2.3. Job Embeddedness Scale (JES)

Mitchell et al. [25] developed this tool in 2001 to assess hospital and chain store employees. In 2005, Liang [34] conducted a cross-cultural adaptation and validation of the tool for use in the employees of private hospitals, with Cronbach's α coefficient of 0.873. This scale has been widely used and validated among Chinese nurses [35], which has 6 dimensions and 37 items: organization fit (7 items), community fit (5 items), organization sacrifice (9 items), community sacrifice (3 items), organization link (7 items), and community link (6 items). Items 1–24 use a 5-point Likert scale (1 = strongly disagree and 5 = strongly agree); Items 25–37 are single-choice questions standardized to Likert scale scores based on the strength of the hypothesized causal relationship (scoring of items differs among regions). For example, for the item “How many years have you been in your current position?,” 1 = “less than one year,” 2 = “1–2 years,” 3 = “3–5 years,” 4 = “6–9 years,” and 5 = “10 years and above.” The total score ranges from 37 to 185, with higher scores indicating a higher level of job embeddedness, and the Cronbach's *α* coefficient for this scale in this study was 0.919.

#### 3.2.4. Psychological Empowerment Scale (PES)

Spreitzer [8] developed it in 1995, and Li et al. [36] modified it according to the Chinese cultural background in 2006. It was used to measure the level of psychological empowerment of Chinese nurses with Cronbach's *α* coefficient of 0.88. The scale has four dimensions and 12 items: meaning (3 items), self-determination (3 items), competence (3 items), and impact (3 items). A five-point Likert scale was used (1 = *strongly disagree* and 5 = *strongly agree*), the total score ranges from 12 to 60, and the higher the score, the greater the psychological empowerment. The Cronbach's *α* coefficient for this scale in this study was 0.919.

### 3.3. Data Collection

**Figure 2:**
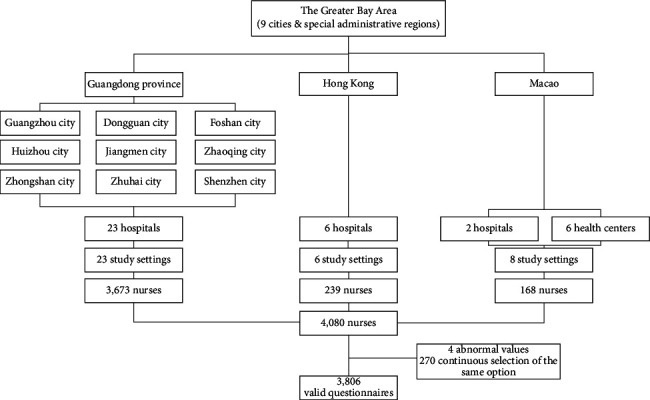
Flow diagram of participant recruitment.

This study, conducted from March 2023 to July 2023, was undertaken with the support of the hospital management departments and nursing colleges in Guangdong, Hong Kong, and Macao. Before data collection, we determined the number of registered nurses in each district by consulting the yearbooks of the regional statistical bureaus. Data from the Guangdong Health Statistics Yearbook 2022 [37], Macao Health Statistics 2022 [38], and Hong Kong Statistics and Lists of Nurses [39] showed that the distribution of nurses in the Greater Bay Area was as follows: 420,790 nurses (88.72%) from Guangdong, 50,650 nurses (10.68%) from Hong Kong, and 2863 nurses (0.60%) from Macao. This information guided us in defining the approximate scope and sample size of the survey for each region, and we used this as a basis for starting data collection in different regions and at different levels of hospitals or health centers. An electronic self-report questionnaire (https://www.wjx.cn) was used to collect data. To ensure the ethical consideration, we set a control for informed consent choices. A total of 4080 participants were recruited to complete the full questionnaire for this study, and after excluding invalid questionnaires (abnormal values and similar options), 3806 valid questionnaires were included for an effective rate of 93.28%. The collection process is shown in Figure 2. We used G^∗^Power [40] to perform a post hoc power analysis for the model with the largest number of variables (Model 4), which included 8 covariates, 7 independent variables (self-efficacy and six dimensions of job embeddedness), number of tested predictors = 7, and total number of predictors = 15. We performed a two-sided test with a large effect size (*f*^2^ = 0.35) [41], setting alpha = 0.05 and sample size N = 3806. Power analysis showed that the power (1-beta err prob) was close to 1, indicating that our model has excellent power.

### 3.4. Statistical Analysis

Data analysis was performed using IBM SPSS Statistics 23.0 and PROCESS V4.1. Continuous variables (self-efficacy, job embeddedness, and psychological empowerment) were described by mean and standard deviation and categorical variables (sociodemographic information) by frequency and percentage. Kurtosis and skewness determine the normality of the data, and if the absolute value of kurtosis is < 10 and the absolute value of skewness is < 3, the data are considered to be analyzed as a normal distribution [42]. Harman's single-factor test was used to test for common method bias, and we used Pearson correlation analysis to examine the relationships between the study variables.

By reviewing possible causal associations reported by published studies on nurses' self-efficacy, job embeddedness, and psychological empowerment (Appendix S1), we constructed a framework of DAG to reveal the MSASs of potential confounders affecting the hypothesized model (Appendix S2). The DAG framework was created and analyzed using DAGitty (https://www.dagitty.net), a browser-based environment for creating, editing, and analyzing causal diagrams, with the aim of using causal diagrams for minimizing bias in empirical research [43].

Multiple linear regression models were used to identify the factors associated with psychological empowerment. Model 1 included the MSASs (gender, age, location, monthly salary level, nature of work organization, specialist nurse, professional title, and working years); Model 2 included the MSASs and self-efficacy; Model 3 included the MSASs, self-efficacy, and job embeddedness; and Model 4 included the MSASs, self-efficacy, and six dimensions of job embeddedness (organizational fit, community fit, organizational sacrifice, community sacrifice, organizational link, and community link).

We used the bias-corrected bootstrapping method to generate 5000 random samples with a 95% bias-corrected confidence interval (CI) to analyze the mediating effect of job embeddedness on self-efficacy and psychological empowerment [44]. Estimating without zero in the 95% CI indicated significant mediation effects. All statistical tests were two-sided, and a *p* value of less than 0.05 was considered statistically significant.

We conducted subgroup analyses based on regions to check whether the mediation effect results of nurses from different regions were significant. In addition, based on the results of region subgroup–mediated models, an appropriate moderated mediation model was used to validate the moderating role of regions [45], and a simple slope test was used to show the interaction between variables [46]. In both analyses, the covariates were the MSASs without regions.

### 3.5. Ethical Consideration

This study received approval from the Institutional Review Board of the Academic Committee at Macao Polytechnic University (NO. RP/AE-06/2022). Before administering the questionnaire survey, the researcher explained the purpose and significance of the study to the potential participants. All participants were informed that their participation was voluntary and that they could withdraw from the study without penalty. It was emphasized that there are no right or wrong answers to the questionnaire, and participants should answer based on their own understanding and feelings to avoid any bias. We assured participants that all collected data would be used solely for academic research purposes, that the questionnaire would be anonymous, and that no personal information would be disclosed, thereby minimizing common methodological biases.

## 4. Results

### 4.1. Participants' Characteristics

Among the 3806 nurse participants, the majority were female (92.72%), and 7.28% were male, 43.35% were ≤ 30 years old, 36.36% were between 31 and 40 years old, 16.47% were between 41 and 50 years old, and only 3.81% were ≥ 51 years old. The majority of respondents (89.60%) were from Guangdong Province, with 6.10% coming from Hong Kong and 4.31% from Macao, similar to the overall distribution of the population. The average working years of nurses was 12.13 ± 8.67, 61.22% of the nurses were married, and slightly less than half had tertiary education or below (49.66%), with 49.24% earning a medium salary. A substantial majority, 76.56%, worked in public hospitals, and 22.15% were specialist nurses. More details are presented in Table 1.

### 4.2. Common Method Bias Test

Due to the use of self-report questionnaires in this study, there is a potential for common method bias in the data. To enhance the rigor of this study, Harman's single-factor test was employed to assess this bias. All items for sociodemographic information, psychological empowerment, self-efficacy, and job embeddedness were included and nonrotated factor analysis was carried out. As results revealed that there are 13 factors with eigenvalues greater than 1, and the first factor accounted for 27.442% of the variance, which is below the critical threshold of 50% [47], therefore, it can be assumed that the data in this study do not suffer from significant common method bias.

### 4.3. Scale Scores and Correlation Analysis

The study found that nurses scored 45.22 ± 6.89 for psychological empowerment, 127.11 ± 19.79 for job embeddedness, and 28.89 ± 4.64 for self-efficacy. The kurtosis of the SES, JES, and PES scores were 1.255, −0.063, and 2.177, respectively, and the skewnesses of these scales were 0.051, −0.143, and −0.491, respectively. We considered that the scale scores all conformed to normal distribution.  Table 2 presents the mean scores for individual dimensions and the results of Pearson correlation analyses for the scales and dimensions of psychological empowerment, job embeddedness, and self-efficacy. Psychological empowerment exhibits a statistically significant positive correlation with both self-efficacy (*r* = 0.650) and job embeddedness (*r* = 0.657), while self-efficacy also demonstrates a statistically significant positive correlation with job embeddedness (*r* = 0.432) (H1). Furthermore, all facets of psychological empowerment and job embeddedness were positively correlated with self-efficacy (all *p* < 0.001).

### 4.4. Factors Associated With Psychological Empowerment

Table 3 presents the results of the multiple linear regression for nurses' psychological empowerment. Model 1 included all covariates and Model 2 added self-efficacy, and the main results are shown in Models 3 and 4. The results of Model 3 (*F* = 377.148, *p* < 0.001, *R*_adj_^2^ = 0.613) showed that self-efficacy (*B* = 0.642, 95% CI: [0.609, 0.675], *p* < 0.001) and job embeddedness (*B* = 0.189, 95% CI: [0.180, 0.198], *p* < 0.001) were positively associated with psychological empowerment (H2). Model 4 (*F* = 351.370, *p* < 0.001, *R*_adj_^2^ = 0.659) further tested the relationship between the dimensions of job embeddedness and psychological empowerment and all dimensions except community link (*p*=0.984) were associated with psychological empowerment (*p* < 0.05).

### 4.5. Mediating Effects of Job Embeddedness on Self-Efficacy and Psychological Empowerment

**Figure 3:**
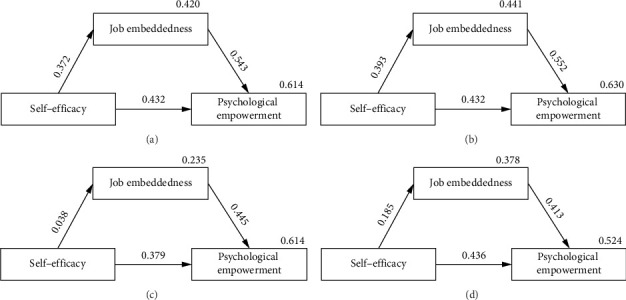
Standardized estimates mediation model. (a) Guangdong–Hong Kong–Macao Greater Bay Area (N = 3806), (b) Guangdong (N = 3410), (c), Hong Kong (N = 232) and (d) Macao (N = 164).

We performed 5000 resamples by bootstrapping to calculate CIs for the mediation effect [44], and the MSASs in the DAG framework were used as covariates in the mediated effects model. Model A showed that the 95% CI of the indirect effect was 0.266–0.335 and that the interval did not include 0, suggesting that job embeddedness did indeed play a mediation effect in the relationship between self-efficacy and psychological empowerment (H3). Self-efficacy had a direct positive impact on psychological empowerment (*B* = 0.642, *p* < 0.001). In addition, self-efficacy played a partial mediation effect in psychological empowerment through job embeddedness (*B* = 0.300, *p* < 0.001). The total effect of self-efficacy on psychological empowerment was 0.942 (*p* < 0.001), with the job embeddedness mediating effect accounting for 31.85% of the total effect (see Table 4). The mediation model explains 46.0% of job embeddedness and 61.9% of psychological empowerment, as illustrated in Figure 3 (covariates not shown).

Furthermore, to assess the significance of the mediation model across different regions, the sample was divided into three groups based on geographical location. Among Guangdong nurses and Macao nurses, job embeddedness mediated 33.51% and 14.90%, respectively, between self-efficacy and psychological empowerment (Model B and Model D). However, among Hong Kong nurses (Model C), job embeddedness did not play a mediating role between self-efficacy and psychological empowerment (*B* = 0.024, 95% CI: [-0.079, 0.150]), and self-efficacy was statistically not significantly associated with job embeddedness (*B* = 0.147, *p*=0.539). For details, refer to Table 4 and Figure 3.

### 4.6. The Moderation Role of Regions

**Figure 4:**
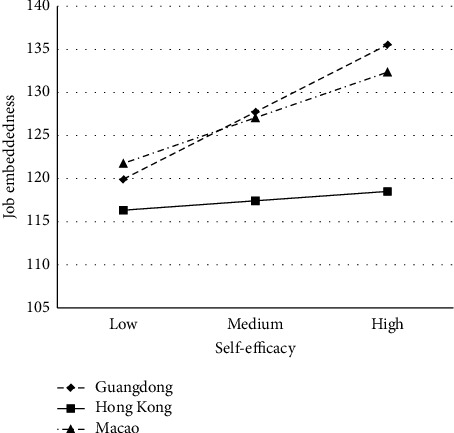
The interaction effect of self-efficacy and regions on job embeddedness.

Based on the mediation effect results for the different regional subgroups, we found that the first half of the mediation model is not significant in Hong Kong. Therefore, we employed the PROCESS macro model 7 to further examine the moderating role of different regions. [45]. The MSASs excluding regions were used as covariates.  Table 5 shows that the interaction between Hong Kong (Guangdong as the reference group) and self-efficacy was statistically significant (*B* = −0.016, *p*=0.004, 95% CI: [−0.026, −0.005]), and the interaction between Macao (Guangdong as the reference group) and self-efficacy was statistically not significant (*B* = −0.541, *p*=0.099, 95% CI: [−1.182, 0.101]). It suggests that the region (between Guangdong and Hong Kong) moderated the relationship between self-efficacy and job embeddedness.  Figure 4 shows the slopes of the relationship between self-efficacy and job embeddedness for nurses in different regions.

## 5. Discussion

This is the first study to investigate the influence of self-efficacy and job embeddedness on psychological empowerment and the mediating role of job embeddedness on the relationship between self-efficacy and psychological empowerment among nurses in the Guangdong–Hong Kong–Macao Greater Bay Area. We emphasized the importance of psychological empowerment in nursing practice, and increasing nurses' self-efficacy and job embeddedness will contribute to psychological empowerment since empowerment only takes effect when nurses actively feel empowered. In addition, the results showed that the association between self-efficacy and job embeddedness was moderated by region, and Hong Kong nurses' self-efficacy did not significantly affect their job embeddedness. The results of this study contribute to further providing a theoretical basis for interventions that enhance nurses' psychological empowerment based on self-efficacy as a pathway.

Our study results indicated that the psychological empowerment score of nurses was 45.22 ± 6.89, which was similar to several other studies in China [48–50]. These studies were conducted among Chinese registered nurses, and the results all showed that nurses' psychological empowerment was at a medium-high level, and it still can be further improved. In addition, the “impact” dimension had the lowest scores of the four dimensions of psychological empowerment, with the same results in studies from different countries and different groups of nurses [48, 51, 52]. The impact is the degree to which an individual can influence strategic, administrative, or operating outcomes at work [53]. “Impact” is influenced by the work context [8]. However, the fact is that in many healthcare organizations, nurses do not seem to have enough influence and power to change their work contexts, and they may be stressed and burned out because of their heavy workloads, lack of leadership support, and lack of work control [54, 55]. Locus of control is the personality trait most associated with the impact dimension and is also associated with empowerment, which refers to the extent to which people determine what happens in their lives in detail on their own rather than by external forces [8]. People with internal locus of control are likelier to feel empowered to shape their work context. Studies have shown that when nurses have work control (decision-making freedom) and autonomy, they have greater independent nursing judgment and experience their work as meaningful. This leads to increased job satisfaction, improved work–family balance, and reduced turnover intentions and intentions to leave nursing [56–58]. Meanwhile, social stereotypes of the professional identity of nursing also diminish the impact of nurses [54]. These distorted stereotypes maintain that nurses are still the handmaidens of the medical staff, a “do” and “female” profession, which affects the identity of nurses and their professional status in society. In addition, students' stereotypes of professional identities become entrenched over time, further contributing to the lack of attractiveness of nursing. This has led to many nurses leaving the profession due to job dissatisfaction and frustration [59]. It is necessary to raise the profile of nursing and place it at the center of health policy [60], develop nurses' leadership skills and empower them to work to their full potential [61], and promote interprofessional education and address stereotypes early in students' professional education [62].

The results showed that self-efficacy had a significant effect on psychological empowerment. In other words, the higher the nurses' self-efficacy, the higher their psychological empowerment. Our study is the first to present and validate this pathway in the nurse population, consistent with the theoretical frameworks. It should be clarified that the concept of psychological empowerment was first defined as the process by which an organization increases employees' self-efficacy through a series of behaviors [17]. However, the self-efficacy concerned by psychological empowerment is preferable to be defined as “competence” rather than “self-efficacy” [18], and this efficacy is defined in the psychological empowerment concept as work-role specific efficacy rather than global efficacy [8]. Thus, we consider self-efficacy and psychological empowerment to be intersecting rather than contained relationships. Nevertheless, the “competence” dimension is translated as “self-efficacy” in the Chinese version of the PES, which may have led to conceptual confusion [36]. In other words, some Chinese studies have interpreted self-efficacy directly as a dimension of psychological empowerment [7, 35], ignoring the fact that self-efficacy is a factor in generating psychological empowerment. Nurses' self-efficacy positively influences psychological empowerment because self-efficacy links organizational-level empowerment interventions to individual-level feelings of empowerment. Empowerment at the organizational level does not consider the feelings of the empowered, so the transferring of power and the intervention of empowerment may not lead to the desired organizational outcome [17]. When organizations empower nurses, increasing nurses' self-efficacy enables nurses to feel more powerful and motivated to complete tasks and thus feel empowered and promote performance. Conversely, when faced with empowerment interventions, nurses with low levels of self-efficacy tend to avoid tasks and scenarios that they perceive to be beyond their capabilities, feel incompetent, and thus cannot feel empowered. This study provides a new way to increase nurses' psychological empowerment. Leaders and nurse managers can help nurses feel empowered by identifying potential contexts that reduce self-efficacy, such as leadership styles, reward and punishment systems, and competitive pressures, and by providing self-efficacy information (such as subjective experiences and positive accomplishments).

We found that job embeddedness and psychological empowerment were positively associated, meaning that the more embedded nurses were in their jobs, the higher were their level of psychological empowerment. This result is similar to the findings of Yoon et al. [63] and Zhou and Chen [64]. Based on cognitive empowerment theory, the outcomes of employee behavior are ambiguous when faced with novel and complex tasks. However, in order to motivate employees to face the task, the outcome of the behavior must be given meaning concerning the individual's goals and behaviors [18]. In other words, if employees do not know what positive or negative outcomes and meanings resulted from completing tasks, they will not be motivated to complete the task and will not feel empowered. Employees want to stay in their jobs because work-related forces capture them in their work, known as job embeddedness [25]. Job embeddedness provides a predictable and objective outcome upon completion of the task, which creates motivation to complete the task, and psychological empowerment. Specifically, when nurses with a higher degree of job embeddedness with their work are confronted with a work task, they can anticipate the impact that completing the task will have on their career development, thus creating motivation to complete the task. Conversely, if nurses are not fit for their jobs, have a weak link to their jobs, or leave their jobs without producing much sacrifice, they will not be motivated to do their jobs, and they will not feel empowered. Such motivation affects nurses' behavior when faced with work tasks, which in turn affects nurses' job embeddedness, creating a continuous cycle. Meanwhile, our study further found that organization fit had the greatest degree of influence on psychological empowerment, suggesting that organization fit is the most important key factor in job embeddedness to enhance nurses' psychological empowerment. The association between community link dimensions and psychological empowerment was insignificant, indicating that nurses' community link does not affect their psychological empowerment, similar to the previous study [65]. Overall, enhancing nurses' job embeddedness, especially organization fit, is essential to improving psychological empowerment, and given the differential effects of job embeddedness dimensions on psychological empowerment, future studies need to further examine the antecedents of the different dimensions of job embeddedness in order to provide more targeted interventions to achieve the projected goals.

This study identified that self-efficacy and job embeddedness have a direct impact on psychological empowerment. In addition, job embeddedness plays a mediation role in the relationship between self-efficacy and psychological empowerment. Therefore, nurses' job embeddedness needs to be strengthened in the intervention that increases nurses' psychological empowerment through self-efficacy pathways. The effect of self-efficacy on psychological empowerment has been reported in previous studies [27, 28]. Kim and Park [28] surveyed 438 nurses in four general hospitals and three small- and medium-sized hospitals in South Korea, and the results showed that nurses who are confident in performing their jobs and tasks have high levels of work engagement and organization embeddedness. Meanwhile, nursing professionalism plays a key role in nurses' self-efficacy and job embeddedness, and when nurses' self-understanding and evaluation are positive, they develop positive professional perceptions and nursing specialization, further improving their job embeddedness. Our study further confirmed that considering self-efficacy only in the empowerment process is imperfect and that employees' perceptions of the characteristics of the job and the socially structured situation in which they work are affected by intrinsic cognitive biases [18]. Specifically, nurses in the process of gaining psychological empowerment not only need to have the confidence to do their work but also need to be embedded in their work. Embeddedness influences their perceptions and attitudes toward their work. Managers should build positive work environments and recognize the importance of decent work that enhances nurses' self-efficacy and optimizes the fit between people and work in the work–life sphere [66, 67]. These strategies will help nurses strengthen their links and fit with their organization. Essentially, nurses will be empowered by the organization, have greater functionality and power in their work, feel more personally valued, and engage with more work and research groups. These will further contribute to the psychological empowerment among high self-efficacy nurses [68].

Given the fact that our study focuses on nurses in the Guangdong–Hong Kong–Macao Greater Bay Area, and that different regions have different cultural and societal backgrounds, which may affect the mediation model, therefore, to further test the difference of the mediation model in different regions, we analyzed the mediation effect on the samples from different regions separately and selected the moderated mediation model based on the results of the subgroup analysis to analyze the moderating effect of regions. The results indicated that the mediating role of job embeddedness in the relationship between self-efficacy and psychological empowerment was significant among nurses in Guangdong and Macao but not among nurses in Hong Kong. The reason is that regions moderated the relationship between self-efficacy and job embeddedness, and Hong Kong nurses' self-efficacy is not associated with their job embeddedness. This is a new finding and different from the results of previous studies [27, 28]. To explain this finding, a Pearson correlation analysis was conducted on Hong Kong nurses' self-efficacy and job embeddedness. The results showed that Hong Kong nurses' self-efficacy had only a low-level positive correlation with the fit (*r* = 0.164, *p* = 0.012) and no significant correlation with link and sacrifice (*r* = 0.035, *r* = 0.025, all *p* > 0.05). Hong Kong nurses' confidence in completing their work does not strengthen their connection to their organization and community and improve their benefits, and the reasons for this result may be related to the local policies and organizational features. Specifically, most nurses working in Hong Kong are locals and thus may have relatively stable organization and community links. In addition, nurses' job entitlements are usually related to professional titles, ranks, and job scope, and nurses with high self-efficacy may not necessarily increase their sacrifice costs, so differences in individual self-efficacy may not be sufficient to influence job embeddedness. Future studies are needed to further examine how Hong Kong nurses are embedded in their work and the antecedents of Hong Kong nurses' job embeddedness. In order to further understand how Hong Kong nurses are embedded in their work, future research should be conducted in a variety of ways, such as through qualitative research methods, to gain insight into why nurses stay in their work hospitals and the factors that facilitate their embedding in their current work. These findings will complement the findings of the quantitative study to further explore the underlying mechanisms that facilitate job embeddedness, empowerment, and retention among Hong Kong nurses.

### 5.1. Limitations

There are several limitations in this study. First, although we identified the covariates of the model through a DAG and used the mediation model for controlling the covariates to validate our hypothesized model, the causal relationship between these factors cannot be inferred from the analysis of the baseline data alone. Future research needs to further identify the causal associations between variables through longitudinal studies, which will help better understand the changing trends in nurses' psychological empowerment and the dynamic associations between variables, ultimately providing more accurate evidence for developing relevant intervention programs. Second, the model we constructed may be influenced by regions with different cultures and policies, which limits the generalization of the results of this study and requires further validation in different countries and regions in the future, as well as the need to explore the incentives for nurses' job embeddedness and psychological empowerment in different cultural contexts. Third, this study only reported Cronbach's *α'*s for the scales and their dimensions; data on the reliability and validity of the scales' other psychometric properties are unknown.

## 6. Conclusion

The study confirmed a pathway for enhancing nurses' psychological empowerment through self-efficacy, grounded in empowerment theory. The psychological empowerment of nurses needs to be further improved, especially regarding their impact, which seems to be underappreciated. Administrators should improve nurses' working environments to increase their influence and decision-making autonomy. The findings indicated that nurses' self-efficacy and job embeddedness directly influence their psychological empowerment, and job embeddedness plays a mediating role in the relationship between self-efficacy and psychological empowerment. Therefore, nursing managers can increase nurses' self-efficacy by identifying work situations that diminish self-efficacy and providing self-efficacy information, thereby increasing nurses' psychological empowerment through this pathway. In addition, it is necessary to consider the nurse's job embeddedness, which provides the nurse with outcomes and goals upon completion of the job and task, thereby increasing the nurse's motivation to complete the task. Future studies need to validate this model further in different regions, obtain more robust causal inferences through longitudinal studies, and develop intervention programs focusing on self-efficacy as a pathway to increase nurses' psychological empowerment.

## Figures and Tables

**Table 1:** Differences in general characteristics among nurses (*n* = 3806).

Demographic characteristics	Frequency	Percentage (%)
Gender		
Male	277	7.28
Female	3529	92.72
Age		
≤ 30	1650	43.35
31∼40	1384	36.36
41∼50	627	16.47
≥ 51	145	3.81
Regions		
Guangdong	3410	89.60
Hong Kong	232	6.10
Macao	164	4.31
Marital status		
Single	1375	36.13
Married	2330	61.22
Divorced	101	2.65
Education		
Junior college and below	1890	49.66
Undergraduate	1696	44.56
Postgraduate and above	220	5.78
Monthly salary level		
Low level	419	11.01
Medium level	1874	49.24
High level	1513	39.75
Nature of work organization		
Public hospital	2914	76.56
Private hospital	732	19.23
Others	160	4.20
Whether or not you have specialist nurse qualifications?		
Yes	843	22.15
No	2963	77.85
Professional title		
Junior title	2215	58.20
Intermediate title	1266	33.26
Senior title	325	8.54

**Table 2:** Reliability, means, standard deviation (SD), and correlation values (*n* = 3806).

Variables	*α*	M	SD	1	2	3	4	5	6	7	8	9	10	11	12	13
1. Psychological empowerment	0.919	3.77	0.57													
2. Meaning	0.856	3.98	0.67	0.837^∗∗^												
3. Self-determination	0.786	3.86	0.64	0.889^∗∗^	0.687^∗∗^											
4. Competence	0.819	3.97	0.59	0.837^∗∗^	0.658^∗∗^	0.742^∗∗^										
5. Impact	0.883	3.26	0.83	0.810^∗∗^	0.512^∗∗^	0.605^∗∗^	0.505^∗∗^									
6. Job embeddedness	0.919	3.44	0.53	0.657^∗∗^	0.620^∗∗^	0.549^∗∗^	0.521^∗∗^	0.523^∗∗^								
7. Organization fit	0.934	3.75	0.72	0.711^∗∗^	0.675^∗∗^	0.625^∗∗^	0.559^∗∗^	0.542^∗∗^	0.802^∗∗^							
8. Community fit	0.939	3.88	0.75	0.550^∗∗^	0.511^∗∗^	0.487^∗∗^	0.480^∗∗^	0.392^∗∗^	0.765^∗∗^	0.634^∗∗^						
9. Organization sacrifice	0.894	3.35	0.69	0.628^∗∗^	0.562^∗∗^	0.528^∗∗^	0.440^∗∗^	0.559^∗∗^	0.805^∗∗^	0.766^∗∗^	0.610^∗∗^					
10. Community sacrifice	0.828	3.58	0.74	0.534^∗∗^	0.480^∗∗^	0.439^∗∗^	0.431^∗∗^	0.444^∗∗^	0.750^∗∗^	0.601^∗∗^	0.716^∗∗^	0.682^∗∗^				
11. Organization link	0.761	3.10	0.74	0.236^∗∗^	0.244^∗∗^	0.162^∗∗^	0.204^∗∗^	0.185^∗∗^	0.602^∗∗^	0.237^∗∗^	0.248^∗∗^	0.203^∗∗^	0.238^∗∗^			
12. Community link	0.577	3.15	0.84	0.200^∗∗^	0.217^∗∗^	0.150^∗∗^	0.173^∗∗^	0.139^∗∗^	0.602^∗∗^	0.215^∗∗^	0.300^∗∗^	0.189^∗∗^	0.277^∗∗^	0.550^∗∗^		
13. Self-efficacy	0.911	2.89	0.46	0.650^∗∗^	0.495^∗∗^	0.575^∗∗^	0.644^∗∗^	0.499^∗∗^	0.432^∗∗^	0.442^∗∗^	0.398^∗∗^	0.403^∗∗^	0.374^∗∗^	0.157^∗∗^	0.132^∗∗^	

*Note: α,* Cronbach's alpha; M, mean.

Abbreviation: SD, standard deviation.

^∗∗^ *p* < 0.001.

**Table 3:** Multiple linear regression analysis model of psychological empowerment among nurses (*n* = 3806).

Variables	Model 1			Model 2			Model 3			Model 4		
	B (95% *CI*)	SE	*p*	B (95% *CI*)	SE	*p*	B (95% *CI*)	SE	*p*	B (95% *CI*)	SE	*p*
Constant	45.491 (43.395, 47.586)	1.069	< 0.001	16.998 (15.060, 18.936)	0.988	< 0.001	4.770 (3.053, 6.486)	0.875	< 0.001	7.145 (5.485, 8.805)	0.847	< 0.001
Gender (male as the reference group)												
Female	−0.610 (−1.474, 0.253)	0.440	0.166	0.155 (−0.508, 0.817)	0.338	0.647	−0.278 (−0.830, 0.275)	0.282	0.324	−0.173 (−0.692, 0.346)	0.265	0.514
Age (≤ 30 as the reference group)												
31–40	0.642 (−0.076, 1.360)	0.366	0.080	0.656 (0.105, 1.206)	0.281	0.020	−0.382 (−0.843, 0.079)	0.235	0.104	0.306 (−0.134, 0.746)	0.225	0.173
41–50	0.008 (−1.244, 1.260)	0.638	0.990	0.166 (−0.793, 1.126)	0.490	0.734	−0.227 (−1.026, 0.573)	0.408	0.578	0.033 (−0.717, 0.784)	0.383	0.930
≥ 51	−0.393 (−2.371, 1.585)	1.009	0.697	0.415 (−1.102, 1.932)	0.774	0.592	1.101 (−0.163, 2.364)	0.644	0.088	0.509 (−0.679, 1.697)	0.606	0.401
Regions (Guangdong as the reference group)												
Hong Kong	−3.813 (−4.896, −2.730)	0.552	< 0.001	−2.607 (−3.439, −1.775)	0.424	< 0.001	−0.703 (−1.401, −0.004)	0.356	0.049	−0.033 (−0.692, 0.627)	0.336	0.922
Macao	−0.553 (−1.708, 0.601)	0.589	0.347	0.631 (−0.255, 1.517)	0.452	0.163	0.690 (−0.047, 1.428)	0.376	0.067	0.957 (0.262, 1.651)	0.354	0.007
Monthly salary level (low level as the reference group)												
Medium level	1.092 (0.367, 1.817)	0.370	0.003	0.717 (0.161, 1.273)	0.284	0.012	−0.221 (−0.686, 0.244)	0.237	0.351	−0.030 (−0.468, 0.407)	0.223	0.893
High level	1.778 (0.972, 2.585)	0.411	< 0.001	0.734 (0.115, 1.354)	0.316	0.020	−0.395 (−0.914, 0.123)	0.264	0.135	−0.080 (−0.571, 0.410)	0.250	0.748
Nature of work organization (public hospitals as the reference group)												
Private hospitals	−0.484 (−1.049, 0.081)	0.288	0.093	−0.342 (−0.775, 0.092)	0.221	0.123	0.549 (0.186, 0.913)	0.185	0.003	0.364 (0.020, 0.707)	0.175	0.038
Others	1.949 (0.796, 3.103)	0.588	0.001	0.842 (−0.043, 1.728)	0.452	0.062	1.237 (0.499, 1.974)	0.376	0.001	1.088 (0.393, 1.783)	0.354	0.002
Whether or not you have specialist nurse qualifications? (yes as the reference group)												
No	−0.777 (−1.341, −0.213)	0.287	0.007	−0.388 (−0.821, 0.044)	0.221	0.078	−0.072 (−0.432, 0.288)	0.184	0.695	−0.157 (−0.498, 0.183)	0.174	0.364
Professional title (junior title as the reference group)												
Intermediate title	0.408 (−0.262, 1.077)	0.342	0.233	0.129 (−0.385, 0.642)	0.262	0.623	−0.184 (−0.612, 0.244)	0.218	0.399	0.127 (−0.276, 0.529)	0.205	0.537
Senior title	1.538 (0.454, 2.621)	0.553	0.005	0.711 (−0.121, 1.542)	0.424	0.094	−0.224 (−0.918, 0.469)	0.354	0.526	0.205 (−0.447, 0.858)	0.333	0.537
Working years	0.068 (0.011, 0.125)	0.029	0.019	0.044 (0.000, 0.088)	0.022	0.050	−0.094 (−0.131, −0.057)	0.019	< 0.001	−0.021 (−0.057, 0.016)	0.019	0.271
Self-efficacy				0.942 (0.906, 0.978)	0.018	< 0.001	0.642 (0.609, 0.675)	0.017	< 0.001	0.585 (0.553, 0.617)	0.016	< 0.001
Job embeddedness							0.189 (0.180, 0.198)	0.005	< 0.001			
Organization fit										0.543 (0.500, 0.586)	0.022	< 0.001
Community fit										0.072 (0.018, 0.126)	0.027	0.009
Organization sacrifice										0.126 (0.090, 0.162)	0.018	< 0.001
Community sacrifice										0.110 (0.017, 0.203)	0.048	0.021
Organization link										0.052 (0.013, 0.091)	0.020	0.008
Community link										0.000 (−0.033, 0.032)	0.017	0.984
*R*^2^	0.053			0.443			0.614			0.661		
Adjusted *R*^2^	0.050			0.441			0.613			0.659		
F	15.166			201.305			377.148			351.370		
*p*	< 0.001			< 0.001			< 0.001			< 0.001		
VIF	1.103–5.368			1.030–5.371			1.104–5.547			1.111–6.170		

*Note*: B, unstandardized coefficient.

Abbreviations: CI, confidence interval; SE, standard error; VIF, variance inflation factor.

**Table 4:** Direct and indirect effects for the mediating effect model.

Model pathways	B	SE	β	95% CI LL	95% CI UL	*p*
Direct effect (self-efficacy ⟶ psychological empowerment)						
Model A	0.642	0.017	0.432	0.609	0.675	< 0.001
Model B	0.643	0.018	0.432	0.609	0.678	< 0.001
Model C	0.541	0.077	0.379	0.390	0.692	< 0.001
Model D	0.617	0.088	0.436	0.444	0.790	< 0.001
Indirect effect (self-efficacy ⟶ job embeddedness ⟶ psychological empowerment)						
Model A	0.300	0.018	0.202	0.266	0.335	—
Model B	0.324	0.019	0.286	0.217	0.360	—
Model C	0.024	0.059	0.017	−0.079	0.150	—
Model D	0.108	0.045	0.076	0.203	0.254	—
Total effect (self-efficacy ⟶ psychological empowerment)						
Model A	0.942	0.018	0.634	0.907	0.978	< 0.001
Model B	0.967	0.191	0.650	0.930	1.004	< 0.001
Model C	0.565	0.086	0.396	0.396	0.734	< 0.001
Model D	0.725	0.094	0.513	0.539	0.911	< 0.001

*Note:* B, unstandardized coefficient; β, standardized effect. Model A: Guangdong–Hong Kong–Macao Greater Bay Area nurses, *n* = 3,806, covariates: the minimum sufficient adjustment sets. Model B: Guangdong nurses, *n* = 3,410, covariates: the minimum sufficient adjustment sets excluding regions. Model C: Hong Kong nurses, *n* = 232, covariates: the minimum sufficient adjustment sets excluding regions. Model D: Macao nurses, *n* = 164, covariates: the minimum sufficient adjustment sets excluding regions.

Abbreviations: CI, confidence interval; LL, lower limit; SE, standard error; UL, upper limit.

**Table 5:** Coefficients for the tested moderated mediation model (*n* = 3806).

Variables	B	SE	*t*	*p*	95% CI LL	95% CI UL
*Model 1 (job embeddedness)*						
Constant	111.230	2.393	46.480	< 0.001	106.538	115.921
Self-efficacy	1.682	0.056	30.246	< 0.001	1.573	1.791
Regions (Guangdong as the reference group)						
Hong Kong	−10.306	1.237	−8.332	< 0.001	−12.731	−7.880
Macao	−0.674	1.341	−0.503	0.615	−3.303	1.955
Self-efficacy × Hong Kong	−1.447	0.230	−6.297	< 0.001	−1.897	−0.996
Self-efficacy × Macao	−0.541	0.327	−1.653	0.099	−1.182	0.101

*R* ^2^	0.427					
F	165.816					
*p*	< 0.001					

*Model 2 (psychological empowerment)*						
Constant	22.797	0.814	27.994	< 0.001	21.201	24.394
Self-efficacy	0.640	0.017	37.922	< 0.001	0.607	0.673
Job embeddedness	0.191	0.005	41.615	< 0.001	0.182	0.200

*R* ^2^	0.613					
*F*	429.535					
*p*	< 0.001					

*Note:* B, unstandardized coefficient, increase of *R*^2^ with interaction: 0.006, *F* = 20.769, *p* < 0.001.

Abbreviations: CI, confidence interval; LL, lower limit; SE, standard error; UL, upper limit.

## Data Availability

The data that support the findings of this study are available from the corresponding author upon reasonable request.

## Supporting Information

Supporting Information 1 Appendix S1: Hypotheses regarding the relationships of exposure, outcomes, and related covariates in the DAGs framework.

Supporting Information 2 Appendix S2: DAGs framework for evaluating the effects of self-efficacy and job embeddedness on psychological empowerment.
