# Supplementary material for: Nurses' Self-Efficacy, Job Embeddedness, and Psychological Empowerment: A Cross-Sectional Study
Source: J Nurs Manag. 2025 Apr 8;2025:6259635. doi: 10.1155/jonm/6259635 (PMC11999754; doi:10.1155/jonm/6259635)
Supplement: Supporting Information 1 — Appendix S1: Hypotheses regarding the relationships of exposure, outcomes, and related covariates in the DAGs framework. [file 6259635.f1.docx]

**Supplementry material 1**

**Appendix S1.**

**Hypotheses regarding the relationships of exposure, outcomes and related covariates in the DAGs framework.**

| **Variables** | **Group** | **DAG paths** | **Reported mechanisms** |
| --- | --- | --- | --- |
| Gender | C | Gender → Self-efficacy | Several studies have shown that male nurses have significantly higher self-efficacy scores than female nurses. (Handiyani et al., 2019) (Simonetti et al., 2021) |
|  |  | Gender → Job embeddedness | Although there was no significant difference in job embeddedness among nurses of different genders in most of the studies (Zhou et al., 2021) (Kim & Kim, 2019), there was still one study that reported a significant difference (Lee & Lee, 2022). Therefore we assume that there is some correlation between these two variables. |
|  |  | Gender → Psychological empowerment | Gender significantly affects the psychological empowerment of nurses. (Permarupan et al., 2019) |
| Age | C | Age → Marital | New nurses have just graduated and have a lower rate of marriage compared to older nurses. |
|  |  | Age → Working seniority | The number of years a nurse has worked increases with age. |
|  |  | Age → Self-efficacy | Age and nurse self-efficacy were positively correlated. (Simonetti et al., 2021) (Ozdemir & Kaplan. 2024) |
|  |  | Age → Job embeddedness | Age is an important influence on job embeddedness, and there was a moderate positive correlation between age and job embeddedness. (Hopson et al., 2018) |
|  |  | Age → Psychological empowerment | Age significantly affects the psychological empowerment of nurses. (Permarupan et al., 2019) (Salles et al., 2020) |
| Region | C | Region → Self-efficacy; Region → Job embeddedness; Region → Psychological empowerment | Although there is no evidence pointed out that there are significant differences in self-efficacy, job embeddedness, and psychological empowerment among nurses in Mainland China, Hong Kong, and Macao. However, due to differences in cultural and political backgrounds, we hypothesized that region would influence nurses' self-efficacy, job embeddedness, and psychological empowerment. |
|  |  | Region → Nature of work organization | There are differences in the nature of hospitals, salary, and title in different regions. |
|  |  | Region → Salary |  |
|  |  | Region → Title |  |
| Marital | C | Marital → Job embeddedness | There is a significant difference in job embeddedness between married and unmarried nurses. (Zhou et al., 2021) |
|  |  | Marital → Psychological empowerment | The level of psychological empowerment of married women is higher than the average. (Abbaszadeh& Boudaghi, 2020) |
| Education | C | Education → Salary | The higher the level of education, the higher the salary package a nurse usually has in the same hospital. |
|  |  | Education → Title | In China, higher education, such as an undergraduate or graduate degree, is usually required for advancement in the title of nurse. |
|  |  | Education → Specialist nurse | Specialty nursing usually requires nurses to have a relatively high level of experience and education. |
|  |  | Education → Nature of work organization | Nurses with different qualifications may choose to work in hospitals of different nature. |
|  |  | Education → Self-efficacy | Significant differences in self-efficacy were found between nurses with bachelor's and diploma degrees. (Soudagar et al., 2015) |
|  |  | Education → Job embeddedness | Differences in job embeddedness among nurses with different educational degrees. (Zhou et al., 2021) (Kim & Kim, 2019) |
| Salary | C | Salary → Self-efficacy | Positive correlation between monthly income and self-efficacy. (Li et al. 2023) |
|  |  | Salary → Job embeddedness | The higher the monthly income the higher the job embeddedness of the nurse. (Zhou et al., 2021) |
|  |  | Salary → Psychological empowerment | Psychological empowerment of nurses differed significantly in different mean monthly income levels. (Cao, 2022) |
| Title | C | Title → Salary | In the same hospital, the higher the title of the nurse, the higher the salary. |
|  |  | Title → Specialist nurse | Nurses usually need a higher title to have the opportunity to become a nurse specialist. |
|  |  | Title → Self-efficacy | Job titles affect nurses' self-efficacy.(Ozdemir & Kaplan. 2024) |
|  |  | Title → Job embeddedness | A study shows that anesthesia nurses' job titles affect their job embeddedness. (Yu et al., 2022) |
|  |  | Title → Psychological empowerment | There was a significant difference in the level of psychological empowerment among nurses with different job titles. (Cao, 2022) |
| Working seniority | C | Working seniority → Title | Nurses need more years of experience to achieve higher titles. |
|  |  | Working seniority → Specialist nurse | Specialty nurses usually require a higher number of years of experience. |
|  |  | Working seniority → Self-efficacy | Years of work experience and self-efficacy were positively correlated. (Gloudemans et al., 2013) |
|  |  | Working seniority → Job embeddedness | Nurses with more years of experience have higher job embeddedness. (Zhou et al., 2021) (Kim & Kim, 2019) |
|  |  | Working seniority → Psychological empowerment | The longer a health professional works in an organization, the more psychological empowerment they become. (Salles et al., 2020) |
| Nature of work organization | C | Nature of work organization → Salary | The nature of the work organization may affect a nurse's salary and title. |
|  |  | Nature of work organization → Title |  |
|  |  | Nature of work organization → Self-efficacy | Private hospital nurses have significantly higher self-efficacy than public hospital nurses. (Handiyani et al., 2019) |
|  |  | Nature of work organization → Job embeddedness | There are differences in the job embeddedness of nurses working in different levels and nature of hospitals. (Cao, 2022) |
|  |  | Nature of work organization → Psychological empowerment | Psychological empowerment is significantly different for nurses in different types of hospitals. (Liu, 2022) |
| Specialist nurse | C | Specialist nurse → Salary | Specialist nurses are usually paid more than general nurses. |
|  |  | Specialist nurse → Self-efficacy | Self-efficacy is a core competency for specialty nurses. (Chen, 2022) |
|  |  | Specialist nurse → Job embeddedness | Although a literature search failed to find evidence that there is a difference between the job embeddedness of specialist nurses and that of general nurses. We hypothesize that specialist nurses have more specialized nursing skills and are more likely to be competent in nursing and therefore may have higher job embeddedness. |
|  |  | Specialist nurse → Psychological empowerment | Specialty nurses usually have higher levels of job autonomy and competence, so although there is no direct evidence that specialty nurses are more psychologically empowered than general nurses, we hypothesize that there is an effect. |
| Self-efficacy | E | Self-efficacy → Job embeddedness | Nurse self-efficacy has a positive impact on job embeddedness. (Kim & Kim, 2019) |
|  |  | Self-efficacy → Psychological empowerment | The study hypothesized that Self-efficacy positively affects psychological empowerment |
| Job embeddedness | E | Job embeddedness → Psychological empowerment | Job embeddedness and psychological empowerment of nurses in county hospitals are positively associated. (Liu, 2020) |
| Psychological empowerment | O | - | - |
| Note: C, Covariate; E, Exposure; O, Outcome | | | |

Abdullatif Ibrahim, I. (2023). Influences of structural empowerment and demographic factors on nurses’ psychological empowerment. Journal of Nursing Management, 2023(1), 8827968. s https://doi.org/10.1155/2023/8827968

Cao J. (2022). A Study on Nurses’ Turnover Intention, Associated Factors and the Pathways to Voluntary Turnover Behavior. (Doctor's thesis, Jilin University).

Chen, X. (2022). Investigation and analysis of influencing factors on core competence of operating room nurses in grade A hospitals of Gansu province. (Master's thesis, Gansu University Of Chinese Medicine)

Gloudemans, H. A., Schalk, R. M., & Reynaert, W. (2013). The relationship between critical thinking skills and self-efficacy beliefs in mental health nurses. Nurse education today, 33(3), 275–280. https://doi.org/10.1016/j.nedt.2012.05.006

Handiyani, H., Kusumawati, A. S., Karmila, R., Wagiono, A., Silowati, T., Lusiyana, A., & Widyana, R. (2019). Nurses’ self-efficacy in Indonesia. Enfermeria clinica, 29, 252-256. https://doi.org/10.1016/j.enfcli.2019.04.030

Hopson, M., Petri, L., & Kufera, J. (2018). A new perspective on nursing retention: Job embeddedness in acute care nurses. Journal for nurses in professional development, 34(1), 31-37. https://doi.org/10.1097/NND.0000000000000420

Kim, H., & Kim, K. (2019). Impact of self-efficacy on the self-leadership of nursing preceptors: The mediating effect of job embeddedness. Journal of nursing management, 27(8), 1756–1763. https://doi.org/10.1111/jonm.12870

Lee, H. J., & Lee, S. K. (2022). Effects of job embeddedness and nursing working environment on turnover intention among trauma centre nurses: A cross‐sectional study. Journal of nursing management, 30(7), 2915-2926. https://doi.org/10.1111/jonm.13666

Li, W., Wan, Z., & XianYu, Y. (2023). Factors influencing nurses self-efficacy two years after the COVID-19 outbreak: A cross-sectional study in Wuhan, China. Medicine, 102(36), e35059. https://doi.org/10.1097/MD.0000000000035059

Liu, Q. (2022). Study on correlation between psychological authorization,job involvement and professional identity ofhemodialysis nurses in 18 tertiary hospitals in Southwest China. Occupation and Health (10), 1370-1374.

Liu, X. (2020). The Research on Embedded and PsychologicalEmpowerment among Nurse in County Hospitals (Master's thesis, Nanjing University of Traditional Chinese Medicine).

Ozdemir, C., & Kaplan, A. (2024). Factors affecting psychological resilience, self-efficacy and job satisfaction of nurse academics: A cross-sectional study. International nursing review, 10.1111/inr.13007. https://doi.org/10.1111/inr.13007

Permarupan, P. Y., Mamun, A. A., Samy, N. K., Saufi, R. A., & Hayat, N. (2019). Effect of psychological empowerment on nurses burnout. The Open Nursing Journal, 13(1). https://doi.org/[10.2174/1874434601913010201](http://dx.doi.org/10.2174/1874434601913010201)

Salles, B. G., Dias, F. C. P., Perissotto, S., Andrade, J. C., Dini, A. P., & Gasparino, R. C. (2020). Psychological empowerment of health professionals. Revista gaucha de enfermagem, 42(spe), e20200050. https://doi.org/10.1590/1983-1447.2021.20200050

Simonetti, V., Durante, A., Ambrosca, R., Arcadi, P., Graziano, G., Pucciarelli, G., Simeone, S., Vellone, E., Alvaro, R., & Cicolini, G. (2021). Anxiety, sleep disorders and self-efficacy among nurses during COVID-19 pandemic: A large cross-sectional study. Journal of clinical nursing, 30(9-10), 1360–1371. https://doi.org/10.1111/jocn.15685

Soudagar, S., Rambod, M., & Beheshtipour, N. (2015). Factors associated with nurses' self-efficacy in clinical setting in Iran, 2013. Iranian journal of nursing and midwifery research, 20(2), 226-231.

Yu, Y., Wei, X. & Chen, J. (2022). The level and determinants of iob embeddedness among nurses in the anesthesiology department. Journal of Nursing Science, (03), 35-38.

Zhou, X., Wu, Z., Liang, D., Jia, R., Wang, M., Chen, C., & Lu, G. (2021). Nurses' voice behaviour: The influence of humble leadership, affective commitment and job embeddedness in China. Journal of Nursing Management, 29(6), 1603-1612. https://doi.org/10.1111/jonm.13306
