# Supplementary material for: Nurses' Self-Efficacy, Job Embeddedness, and Psychological Empowerment: A Cross-Sectional Study
Source: J Nurs Manag. 2025 Apr 8;2025:6259635. doi: 10.1155/jonm/6259635 (PMC11999754; doi:10.1155/jonm/6259635)
Supplement: Supporting Information 2 — Appendix S2: DAGs framework for evaluating the effects of self-efficacy and job embeddedness on psychological empowerment. [file 6259635.f2.docx]

**Supplementary material 2**

**DAGs framework for evaluating the effects of self-efficacy and job embeddedness on psychological empowerment.**


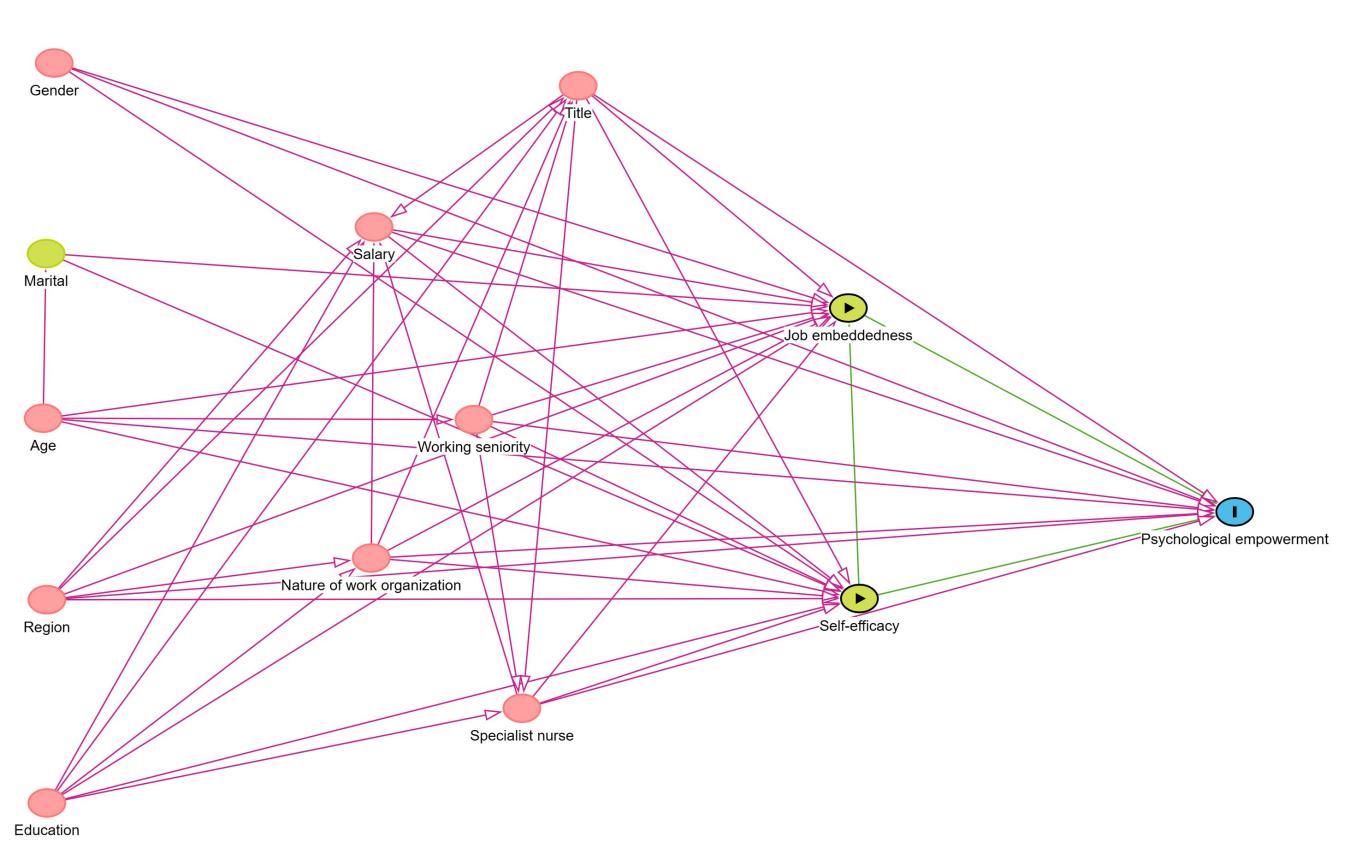


**a. Potential confounders**


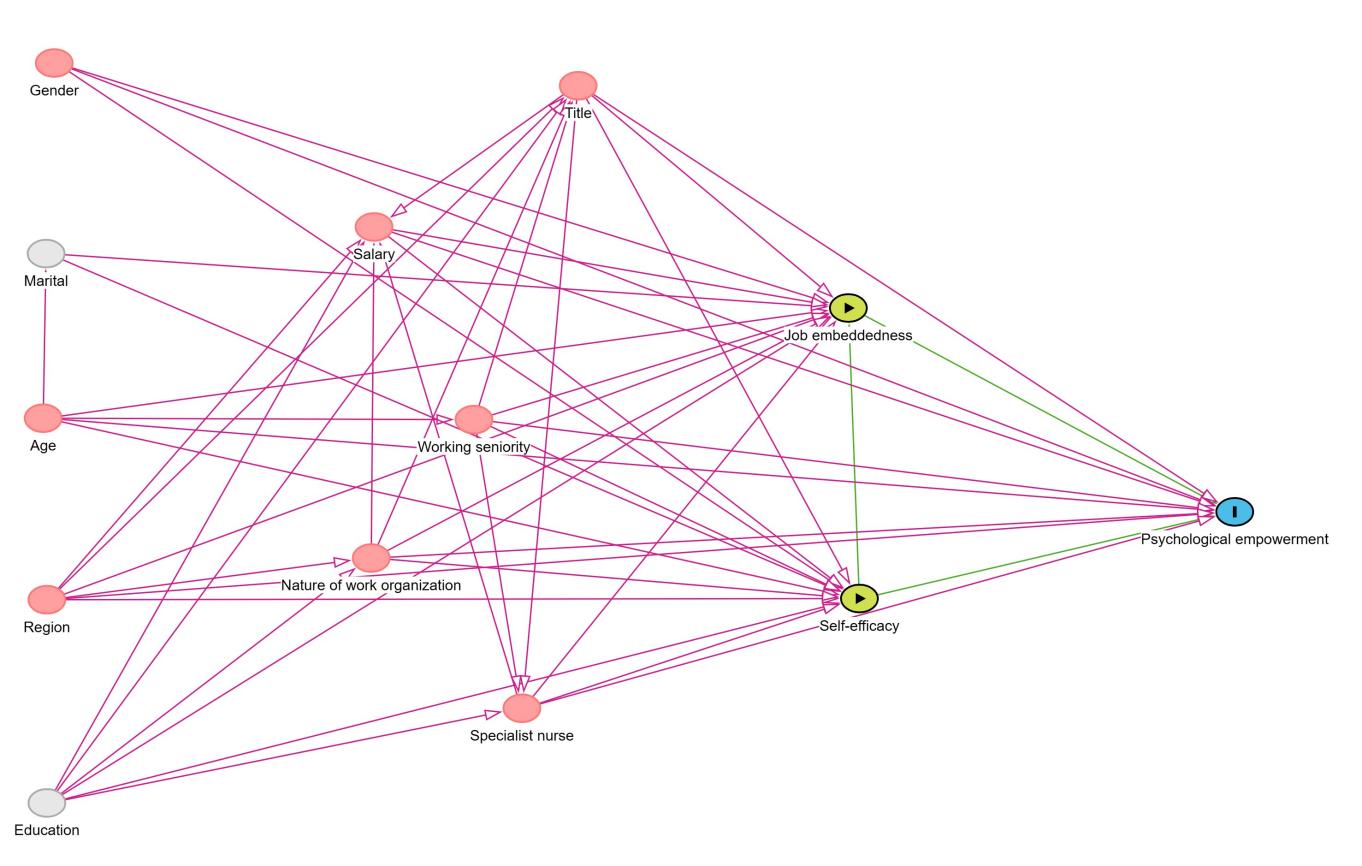


**b. Minimal sufficient adjustment sets**


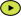
 exposure


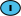
 outcome


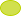
 ancestor of exposure


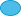
 ancestor of outcome


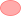
 ancestor of exposure and outcome


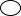
 adjusted variable


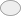
 unobserved (latent)


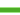
 causal path


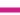
 biasing path

Exposures: Job embeddedness,Self-efficacy

Outcome: Psychological empowerment

**Biasing paths are open.**

Minimal sufficient adjustment sets for estimating the total effect of Job embeddedness, Self-efficacy on Psychological empowerment: Age, Gender, Nature of work organization, Region, Salary, Specialist nurse, Title, Working seniority
